# Role of connectivity anisotropies in the dynamics of cultured neuronal networks

**DOI:** 10.1371/journal.pcbi.1012727

**Published:** 2025-11-06

**Authors:** Akke Mats Houben, Jordi Garcia-Ojalvo, Jordi Soriano

**Affiliations:** 1 Departament de Física de la Matèria Condensada, Universitat de Barcelona, Barcelona, Spain; 2 Universitat de Barcelona Institute of Complex Systems (UBICS), Barcelona, Spain; 3 Department of Medicine and Life Sciences, Universitat Pompeu Fabra, Barcelona, Spain; University of Edinburgh, UNITED KINGDOM OF GREAT BRITAIN AND NORTHERN IRELAND

## Abstract

An inherent challenge in designing laboratory-grown, engineered living neuronal networks lies in predicting the dynamic repertoire of the resulting network and its sensitivity to experimental variables. To fill this gap, and inspired by recent experimental studies, we present a numerical model designed to replicate the anisotropies in connectivity introduced through engineering, characterize the emergent collective behavior of the neuronal network, and make predictions. The numerical model is developed to replicate experimental data, and subsequently used to quantify network dynamics in relation to tunable structural and dynamical parameters. These include the strength of imprinted anisotropies, synaptic noise, and average axon lengths. We show that the model successfully captures the behavior of engineered neuronal cultures, revealing a rich repertoire of activity patterns that are highly sensitive to connectivity architecture and noise levels. Specifically, the imprinted anisotropies promote modularity and high clustering coefficients, substantially reducing the pathological-like bursting of standard neuronal cultures, whereas noise and axonal length influence the variability in dynamical states and activity propagation velocities. Moreover, connectivity anisotropies significantly enhance the ability to reconstruct structural connectivity from activity data, an aspect that is important to understand the structure-function relationship in neuronal networks. Our work provides a robust in silico framework to assist experimentalists in the design of in vitro neuronal systems and in anticipating their outcomes. This predictive capability is particularly valuable in developing reliable brain-on-a-chip platforms and in exploring fundamental aspects of neural computation, including input-output relationships and information coding.

## Author summary

The computational modeling of living neuronal networks has become an important tool to help design experiments, test analytical methods and make predictions. Based on the growing interest in engineering neuronal circuits in vitro, here we provide a numerical model of neurons growing in anisotropic substrates, where neurons and connections are guided to grow in specific regions or follow pre-established paths. The model combines an algorithm to obtain biologically realistic connectivity, together with the Izhikevich neuronal dynamics to replicate the collective behavior of neuronal networks. The model reproduces well experimental observations and is able to make important predictions, such as the impact of development or synaptic noise on network dynamical traits. The model also demonstrates that the presence of anisotropies, and inherited constraints in neuronal connectivity, facilitates the reconstruction of structural connectivity from dynamics, a feature that is important to help designing in vitro systems aimed at approaching brain architectural and dynamical traits.

## Introduction

*In vitro* cultured neurons are a widely used experimental model system to study the dynamics of complex networks of active excitable elements [1,2]. The accessibility and ease of manipulation of neurons in culture allow the *ad hoc* design of different network configurations and physicochemical interventions, while monitoring single cell behavior in relation to emergent collective properties [3,4]. This malleability and observation potential enables to study questions such as plasticity [5–7], signal processing and propagation [8–10], the interchange between segregated and synchronized activity [11,12], and the dynamical changes under pathological states [13,14], up to more abstract phenomena such as learning and memory [15–17]. The versatility and range of applicability of neuronal cultures have favored their extensive use to model universal phenomena in laboratory living neuronal circuits and the brain [3,4,18].

However, cultured neuronal networks typically display a relatively poor dynamical behavior, such as dichotomous dynamics characterized by culture-wide coherent activity events (*network bursts*) interspersed with sparse random activations [19–21]. Neurons and astrocytes are an ensemble of dissociated cells at the time of plating and over time develop into a *de novo* network [4], following in part the developmental trajectory of *in vivo* networks [22–24], yet devoid of most of the guiding factors present in the living brain. Already within 24 hours after plating, the neurons start to extent neurites [18,25,26] in order to form connections to other neurons, however connections are reliably formed only after 7 days in vitro (DIV) [25,27]. Within the same time frame the astrocytes typically proliferate to cover the whole plating substrate [25]. Along this first week of development, neurons predominantly activate in an uncorrelated manner [26,28], with the number of firing neurons increasing with time [28–31]. In the second week after plating, neurons start to display correlated activity [23,26,28,29,32] in the form of fast propagating waves of activity, termed *network bursts*, interspersed with periods of low activity. Along development *in vitro*, the mean number of synapses per neuron increases up until 4 weeks after plating [27]. These changes are reflected in the activity of the neuronal cultures by an increase in the propagation speed of the activity wave [26,30]. The duration of the high activity periods initiated by the initial propagating wave changes along development with an initial increase in duration up until about DIV 28, followed by a shortening of the average network burst duration [26,29,33], but accompanied by an increase in the variability of network burst durations [29]. Interestingly, the activity patterns contained in the network bursts are highly conserved along time [26,28,32,33], indicating that the specific small-scale activity patterns are determined by the structure of the networks.

Whereas the activity of neuronal networks in the mature living brain is constantly driven by internal as well as external (sensory) inputs, the early stages of development *in vitro* occur largely in a state of sensory deprivation, and are hence dominated by spontaneous activity [34]. Spontaneous activations shape the structure of *in vitro* networks along development [25,35], but are also crucial for the correct development of *in vivo* networks [22,24,34–36], guiding neurite growth and modulating neuronal apoptosis. Just before the natural birth date, spontaneous correlated calcium transients between populations of neurons can already be recorded [24], followed by large scale waves of activity propagating across the whole cortex [24,34]. During the first post-natal weeks, across different brain regions, large recurrent network-driven synaptic events separated by periods of sparse uncorrelated activity start to dominate the activity [22,24,34,36], much like the activity recorded in *in vitro* cultured neurons. In the living developing brain, these synapse-driven collective activations are thought to be crucial to develop functional ensembles [22,24,34–37]. Upon birth, cortical activity switches from large-scale synchronous to sparse de-synchronized activity, in part due to increased sensory driven input [34,38], with varying sizes of co-activating ensembles [39]. Yet, de-correlated input alone is not sufficient to drive *in vitro* cultures to an asynchronous activity state, but some degree of structural traits, such as modularity, is needed [12].

In summary, *in vitro* neuronal cultures display activity reminiscent of the early stages of (cortical) development, but they lack the rich dynamics of later developmental stage and mature networks [39], rather resembling pathological states like epilepsy [14]. To enrich network dynamics *in vitro*, micro-engineered structures can be used to restrict and guide the connections between subpopulations of neurons, imprinting anisotropies such as modular organization and directed connectivity that promote the emergence of more diverse activity patterns [3,11,12,40–43].

In a recent work by Montalà-Flaquer et al. [44], polydimethylsiloxane (PDMS) topographical patterns were used to create an inhomogeneous growth environment that introduced subtle modulations in axon growth directions. The advantage of such mild modulations to realize modular networks in a bottom-up manner, as compared to strict confinement of neurons and connections, is that topographical modulations balance a coarse guidance of connections with network-wide self-organization, thus promoting the development of mesoscopic architecture without imposing rigid microscopic blueprints, a concept that was initially explored in Refs [45,46]. In the study of Montalà-Flaquer et al. [44], the topographical shapes came in two variants: (i) consistently interspaced parallel tracks and (ii) randomly placed squares. The introduction of these patterns infused the neuronal cultures with more diverse spontaneous activity repertoires, resembling those of neuronal circuits in the active brain. A key feature of these subtly modulated patterns is that they facilitate local connectivity without fully suppressing network-wide activity, leaving the integration-segregation capacity of the neuronal networks [11] intact.

The above-described ability to modulate and guide the formation of connections in neuronal cultures opens up a vast array of experimental and engineering possibilities. However, experiments take time to plan and execute, so it would be advantageous to be able to use an *in silico* model to quickly explore possible PDMS structures or experimental manipulations. In addition, having a numerical model of inhomogeneous neuronal cultures also allows to manipulate aspects which are difficult to control in biological neuronal cultures, and gives insight into aspects that are not directly observable experimentally, most prominently the structural connectivity of the neurons. In that way, having access to a computational model aids to better understand phenomena observed in experiments.

Following this line of reasoning, in this paper we present a numerical model of neuronal cultures grown in topographically patterned environments. First we show that the model agrees with existing experimental observations [44]. Next, the numerical simulations are used to (i) investigate the relationship between structural connectivity and dynamics, (ii) elucidate the effects of spatial anisotropy and noisy driving on the dynamics of the neuronal cultures, and (iii) to shed light on the ease of reconstructing the connectivity from recorded spontaneous activity under different conditions.

## Results

**Fig 1:**
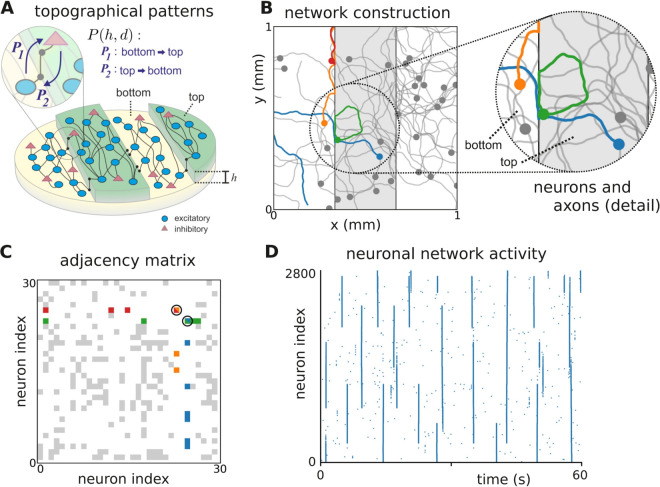
*In silico* construction of neuronal networks with topography. **(A)** Conceptual representation of a topographical network, in which a mixed population of excitatory and inhibitory neurons connect following the tracks at the bottom or at the top of a profile of height *h*. The axons of neurons may pass into other tracks with probabilities *P*_1_ and *P*_2_. **(B)** Illustrative network layout as implemented by the *in silico* model. Only 30 neurons are shown for clarity. Colored dots and lines illustrate the location of neuronal bodies and the excursions of their axons. The blue axon crosses from top to bottom, while the orange, red and green follow the edges of the pattern. **(C)** Corresponding connectivity matrix, highlighting the interconnectivity between the colored neurons. **(D)** Resulting network activity displayed as a rasterplot, in which each blue dot indicates the activation of a neuron (indexed along the vertical axis) along time (horizontal axis).

We carried out an *in silico* exploration of the growth and activity of neuronal cultures in inhomogeneous environments. The inhomogeneities consisted of topographical modulations of height *h* in the substrate where neurons sit, illustrated in Fig 1. As described in Methods, the *in silico* approach consists of two stages. First, the neuronal connections are established by simulating axon growth in two-dimensions whilst modeling the effect of PDMS obstacles on the direction of axon growth (Fig 1A and 1B). This stage determines the network connectivity matrix (Fig 1C). Secondly, spontaneous activity on the generated neuronal networks is simulated using the Izhikevich model (with 80% excitatory neurons and 20% inhibitory ones), and the obtained spike times for the entire network population (Fig 1D) are stored for further analysis. Details on the axon growth algorithm and neuronal model are given in the Methods.

The parameters governing the neuronal dynamics were set such that the flat *Control* condition, i.e., with *h* = 0, reproduced qualitatively the dynamics observed in experiments on a flat surface [44], characterized by strong network bursting events in which all neurons activated together in a short time window, or remained silent. The effect of *h* > 0 on the crossing probabilities was fitted to experimental data [47] that quantified the likelihood that neuronal activity occurring at an area with *h* = 0 could extend to another area with *h* > 0, as described in the Methods. The crossing probabilities are given in Table 1, and the resulting parameter values for the neuronal dynamics are listed in Table 2.

**Table 1:** Probabilities of activity and axons crossing a PDMS border.

PDMS height h (mm)	Activity propagation		Axon crossing	
	bottom→top	top→bottom	$P_{\text{bottom}\to \text{top}}$	$P_{\text{top}\to \text{bottom}}$
0.0	1	1	1	1
0.1	0.60	0.85	$0.45\times 10^{-3}$	$3.3\times 10^{-3}$
0.4	0.15	0.90	$0.25\times 10^{-3}$	$3.3\times 10^{-3}$
0.6	0.05	0.30	$0.02\times 10^{-3}$	$0.50\times 10^{-3}$
>0.7	0	0	0	0

**Table 2:** Parameter values used for simulations.

Parameter	value	unit	
Neuron dynamics			
*σ*		${\text{mV}}^{2}\text{ms}$	noise intensity
*ε*	0.02	ms	adaptation time-scale
*ρ*	0.2		adaptation *v*-sensitivity
$v_{c}$	30	mV	voltage threshold
$v_{0}$	–65	mV	voltage reset value
$\delta_{u}$	6.5		adaptation reset increment
Synapse dynamics			
$\tau_{p}^{E}$	10	ms	excitatory synaptic time-scale
$\delta_{p}^{E}$	3	mV	max. excitatory post-synaptic potential
$\tau_{p}^{I}$	10	ms	inhibitory synaptic time-scale
$\delta_{p}^{I}$	–6	mV	max. inhibitory post-synaptic potential
$\tau_{q}$	$1\times 10^{3}$	ms	synaptic depression time-scale
$\delta_{q}$	0.8		decrease of synaptic vesicle pool

Unless stated otherwise, the numerical results presented below include 10 independent, 600-second-long simulations for each condition and parameter value. Where appropriate, statistical tests have been carried out to understand differences between conditions and parameters for various measures. Figures include graphical indications of the significance of statistical tests and the comparisons carried out. All comparisons, test statistics and p-values can be found in the supporting information S1 Appendix.

### Dynamics of homogeneous and anisotropic *in silico* cultures

**Fig 2:**
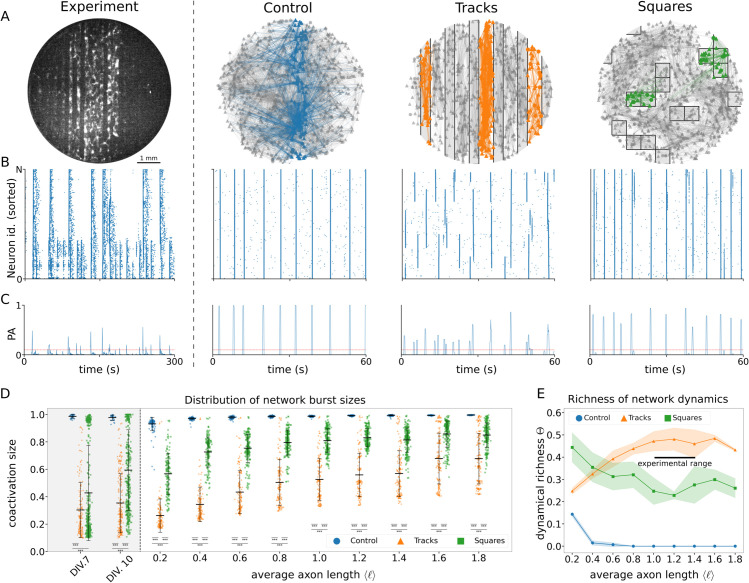
Spontaneous activity in homogeneous and topographically-patterned networks. **(A)** Left panel shows a fluorescence image of an *in vitro* neuronal network, with bright objects corresponding to active neurons. The remaining panels show examples of simulated neuronal culture layouts and connectivity. In the simulation panels, triangles correspond to excitatory and circles to inhibitory neurons. Only 25% of neurons and their connections are shown for clarity. For each simulated condition a group of neurons and their outgoing connections are highlighted to illustrate the effect of the PDMS obstacles. **(B)** Raster plots of the networks in (A). Each dot represents a spike of a neuron. Neurons are sorted by their position along the left-right direction, with the leftmost neuron at the bottom (0) and the rightmost neuron on the top (*N*). Time is represented along the horizontal axis. In the first and third columns, corresponding to the *Tracks* condition in experimental and simulated data respectively, the background shading separates the neurons into the different tracks. **(C)** Population activity (*PA*) plots showing the summed activity traces corresponding to the raster plots of panel B. The value ‘1’ corresponds to the full culture being active in a short time window. The red line indicates PA=0.1, which we use as a threshold to accept network bursts as significant (as compared to background activity) throughout the paper. Only 1-minute sections of full simulations are shown for clarity. **(D)** Distribution of the sizes of network bursts along development for different conditions. For each value of ⟨ℓ⟩, indicated along the *x*-axis, the peaks of the summed activity traces (see panel C) are plotted as points. The two gray-shaded plots on the left correspond to experimental results from [44]. For each axon length the peaks are plotted for the three conditions next to each other: *Control* (blue circles), *Tracks* (orange triangles) and *Squares* (green squares). The figure shows network burst sizes of ten independent simulation runs, each with a length of 5 minutes of simulation time, for varying axon length ⟨ℓ⟩ and for the three experimental setups shown in (A). Stars indicate significance of Mann-Whitney-Wilcoxon tests as follows: *: *p* < 0.05; **: *p* < 0.01; ***: *p* < 0.001. **(E)** Richness of network activity shown in (D) for different axon lengths and setups. Each line corresponds to a single condition: *Control* (blue), *Tracks* (orange) and *Squares* (green). Each dot indicates the average over 10 independent simulations runs, each with a length of 5 minutes. Free simulation parameters: $\sigma =2.00\;{\text{mV}}^{2}/\text{ms}$, h=0.1 mm, and in addition for panels A-C ⟨ℓ⟩=1 mm.

In order to validate the presented model, the simulated activity is compared to previously published experimental results. Fig 2 summarizes the network connectivity and resulting activity dynamics of the model in the three conditions studied experimentally in [44] (*Control*, *Tracks* and *Squares*), rendering it possible to compare the numerical simulations to experimental data. The first column of Fig 2A–2C provides an example of the characteristic results obtained in biological cultures grown on PDMS tracks, showing a snapshot of the dynamics of the network together with the raster plot and the population activity (PA), i.e., the fraction of active neurons within a small time window. In the plots one can observe that the dynamics encompasses different groups of highly coordinated neurons, from small assemblies to the entire network, in contrast with a standard culture grown on a flat surface in which all neurons activate together or remain silent otherwise [44].

In line with the experimental observations, numerical simulations show that, in a flat growth environment (*Control* condition; second column of Fig 2A–2C), axon positioning is unrestricted by obstacles. As such, neurons are able to establish a dense patchwork of connections, and can have long range projections that may reach the full width of the culture. This leads to culture-wide synchronized activity interspersed with periods of low activity (Fig 2B and 2C), as is characteristic for standard, unperturbed neuronal cultures [19,20,44].

In contrast, in the *Tracks* condition (third column of Fig 2A), the obstacles lead to an anisotropic connectivity profile with abundant long-range connections along the tracks and few short-range connections traversing them. From this we expect that a track can be seen as a densely connected neuronal module that is weakly coupled to its neighboring tracks. Indeed, from the raster plot (Fig 2B, third column) we observe that the periods of synchronized activity now consist of groups of neurons with a large variability in sizes: either single tracks, several temporarily synchronized tracks, or the full culture, are contemporaneously active. The network population activity (Fig 2C) highlights this diversity of co-activation sizes. The activity dynamics of the *Tracks* condition are indeed fitting to that of weakly interdependent modules. Each module seems to follow a dynamics similar to that of the full *Control* culture, albeit at a smaller scale. We note that the modules sporadically synchronize with each other, leading to larger network bursts and a resetting of the phases of the synchronized tracks.

Lastly, the *Squares* condition emerges as an intermediate state between the *Control* and *Tracks* cultures in terms of structural connectivity. Indeed, a large area exists in which axon growth is unobstructed, leading to a large group of neurons that can interconnect as in the *Control* condition. However small patches of largely isolated neurons constitute separate modules, weakly connected to the large interconnected group and potentially to other modules. This connectivity, illustrated in the last column of Fig 2A, leads to activity dynamics that is also intermediate between the *Control* and *Tracks* conditions, as expected. This is clear by comparing, e.g., the second and third columns with the last column of Fig 2B and 2C. In most of the cases, synchronized activity encompasses the whole culture, or a large fraction of it. Occasionally, the neurons of one or several of the largely isolated modules collectively activate in isolation from the rest of the culture.

#### Network development.

In the experimental study it was observed that along development (number of days *in vitro* (DIV) since plating the neurons on the glass cover slips), the distribution of network burst sizes changed in a distinct manner for the different conditions [44]. From the first day that activity was detectable, the *in vitro Control* cultures tended to synchronize fully during each network burst, a highly rigid dynamics that was observed until the last recording day (blue dots in the gray-shaded plots of Fig 2D). In contrast, the *Tracks* and *Squares in vitro* cultures both exhibited a wider distribution of burst sizes, that changed along development. As shown in the gray-shaded plots in Fig 2D, the *in vitro Tracks* condition started off at DIV 7 with a distribution heavily biased towards small numbers of neurons co-activating, which broadened to also include larger groups of co-activating neurons at the more developed stage DIV 10. The *in vitro Squares* condition plays an intermediate role, with a nearly bimodal distribution for young cultures at DIV 7 displaying small bursts alternating with large culture-wide bursts. For older *in vitro Squares* cultures at DIV 10, the small bursts disappeared, showing mainly intermediate to large co-activation sizes.

In light of these results, it was proposed [44] that one main aspect governing the development of neuronal cultures after plating is the growth of axons, leading to a gradual increase in the amount and range of neuronal projections and, therefore, a higher probability for a neuron to connect with any other in the network. In the experiments with track modulations of Ref. [44], it was observed that connectivity along tracks in mature cultures at DIV 14 was about 4 times higher than across tracks. Since axonal growth along the tracks is unrestricted, whereas across tracks it is strongly constrained by the track spacing of 200+300 *μ*m, one can argue that axons along tracks can easily reach distances of 0.5×4=1 mm. This value is in agreement with the independent experimental observations by Feinernam *et al.* in uni-dimensional neuronal cultures (conceptually a very long single track) [9], and therefore we can consider in our numerical model that axons about 1 mm long are representative of a mature network stage. Thus, as a proxy to model network development in the numerical simulations presented here, simulation runs were carried out for progressively higher values of the parameter ⟨ℓ⟩ that determines the average axon length during the network growth phase. S1 Fig shows the average degree per neuron and average connection length with increasing axon lengths ⟨ℓ⟩. The distributions of network burst sizes for the three network conditions and different values of ⟨ℓ⟩ are shown in Fig 2D. For the *Control* and *Tracks* conditions, the distributions show a development qualitatively similar to the experimentally observed distributions, with the *Control* condition already displaying culture-wide synchronization for very short average axon lengths ⟨ℓ⟩=0.2 mm, and the *Tracks* condition starting off concentrated at small co-activation sizes with a widening of the distribution for increasing ⟨ℓ⟩. The *Squares* condition shows intermediate distributions between the *Control* and *Tracks* conditions, although the bimodal character of the distribution seen in experiments is not observed in the numerical simulations. This is possibly due to the fact that, in the experiments, a small group of isolated neurons may strengthen their connections to spontaneously activate, a phenomenon mediated by complex plasticity mechanisms that were not included in the simulations. By fitting a linear model to predict the burst sizes from the average axon length ⟨ℓ⟩, condition, and their interaction, it was found that the interaction between axon length and condition was significant (*p* < 0.001), hence indicating that the effect of axon length on the burst sizes differs per condition. For the *Control* condition the slope of ⟨ℓ⟩ was 0.0292, for *Tracks* this increased nearly tenfold to 0.247, whereas for *Squares* it only increased to 0.130. These results highlight the pronounced effect of the *Tracks* substrate on network bursting. The complete model output is provided in S1 Appendix.

An alternative representation of the distribution of co-activation sizes consists in summarizing the burst sizes for each simulation run into a single number that captures the ‘dynamical richness’ Θ of the activity. This measure was proposed and used to quantify the difference in activity patterns for different neuronal culture setups [11]. The computation of Θ from the raster plot data is described in the Methods section. For the simulated data, the dynamical richness, shown in Fig 2E, displays interesting dependencies on the average axon length ⟨ℓ⟩, distinct for each condition. The *Control* and *Squares* conditions exhibit their dynamically richer activity for short axon lengths ⟨ℓ⟩=0.2 mm, with the value Θ decreasing monotonically for increasing ⟨ℓ⟩. In the *Control* condition Θ vanishes for short average axon lengths ⟨ℓ⟩≈0.8 mm, since each coactivation encompasses the whole network already for short axon lengths. The *Squares* condition has an overall higher dynamical richness because of the presence of isolated modules, which cause variation in the number of neurons that coactivate in each network burst. The *Tracks* condition displays a non-monotonic relation between dynamical richness Θ and ⟨ℓ⟩, with optimal dynamically rich activity for intermediate average axon lengths ⟨ℓ⟩ and decreasing Θ on both extremes. This behavior can be interpreted as resulting from the large connectivity anisotropy of the system. This anisotropy leads to rich dynamics when the average axon length is large enough to excite multiple neurons within the same track, but not too large as to consistently induce collective activity in multiple tracks simultaneously. The results can be compared to the reference experimental work [44], in which it was shown Θ was maximal between 10 and 14 DIV. Thus, as highlighted in Fig 2E, ‘mature’ *in silico* networks approximately correspond to axon lengths in the range [1,1.4] mm.

#### Initiation and propagation of fronts.

**Fig 3:**
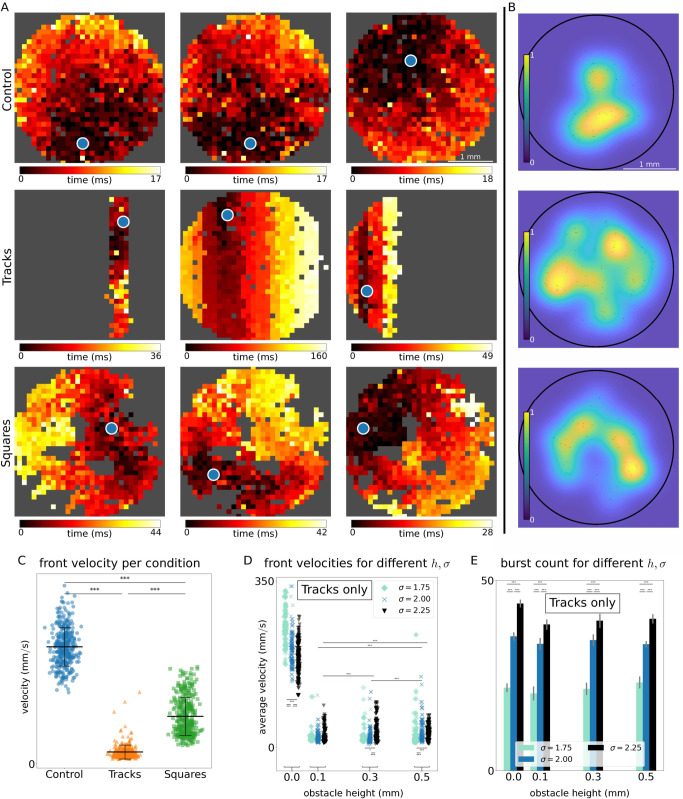
Front propagation in different conditions. **(A)** Representative activity propagation through the neuronal cultures under different conditions. Color-coding indicates first activation time of neurons in the spatial grid point within the front. Start of the activity wave is indicated with a blue dot. **(B)** Spatial distribution of network burst initiation points (black dots) and its probability density function (pdf, blue-yellow colormap) for the different conditions as indicated on the left of panel (A). **(C)** Activity propagation velocities of each network burst for the different conditions. Stars indicate significance of Mann-Whitney-Wilcoxon tests as follows: ***: *p* < 0.001. **(D)** Network burst propagation velocities for different parameter values of the obstacle height *h* and noise intensity *σ* for the *Tracks* conditions. Stars indicate significance of Mann-Whitney-Wilcoxon tests as follows: *: *p* < 0.05; **: *p* < 0.01; ***: *p* < 0.001. **(E)** Barplot indicating average number of network bursts for the same parameter values as in panel (D). Gray lines indicate the standard deviation across simulation runs. Stars indicate significance of Mann-Whitney-Wilcoxon tests as follows: *: *p* < 0.05; **: *p* < 0.01; ***: *p* < 0.001. For each panel ⟨ℓ⟩=1 mm. For panels A-C σ=2.00 and h=0.1 mm.

We have seen so far that the activity of neuronal cultures is characterized by network bursts. From a spatiotemporal point of view, a network burst consists of a propagating activity front, successively exciting neurons such that each neuron fires multiple spikes in a short time span, followed by a phase-waveback, in which each neuron falls silent due to the depletion of synaptic resources. As such, it is illuminating to investigate the spatial propagation of activity fronts by considering the first activation time of each neuron within the burst (Fig 3A), the burst’s initiation points (Fig 3B), and propagation velocities (Fig 3C).

Fig 3A shows representative examples of propagating bursts for the *Control*, *Tracks* and *Squares* conditions. A visual inspection of the patterns reveal that the details of activity propagation, as well as the area covered in each burst, differs across designs. In the *Control* condition (first row in Fig 3A) activity propagates as uninterrupted quasi-circular fronts that rapidly cross the whole culture, which agrees with the observation that each burst encompasses the full network. For the *Tracks* condition (second row in Fig 3A), and as discussed before, the dynamics is reminiscent of a set of transiently synchronizing weakly coupled modules, each module behaving as a stochastic oscillator. As such it is expected that for some network bursts, activity only propagates across a single track (first plot in row) or a low number (third plot in row) of them, and at other times that the activity propagates throughout the whole culture (second plot in row). Moreover, when the burst traverses several tracks, the activity propagates first on a short time-scale within each track (as shown by each track having almost the same color in the second and third plots of the row), passing subsequently on to the next track on a slower time-scale (indicated by the fact that each track has a different color in the plot). Due to this, the total duration of each network burst is dependent on the spatial scale of the activity propagation. Activity propagating within one track or a low number of tracks takes much less time (first and thirds plots in row), than a front traversing the whole culture (second plot in row), as seen by different ranges of the colorbars in the three plots. Lastly, the activity propagation in the *Squares* condition (third row in Fig 3A) is similar to that of the *Control* condition, yet the propagation reveals intricate paths around the PDMS obstacles. This intricacy emerges from the fact that propagation can occur within each square module, on the main surface, or switching from one to another, leading to long, serpentine-like excursions across the culture.

In addition to front propagation, one can also study the distribution of burst initiation points, i.e., the Euclidean position in the culture where each burst originated. This analysis is shown in Fig 3B where, for clarity of representation, the distribution of initiation points is shown as a blue-yellow heatmap that portrays the initiation probability. In accordance with previous experimental results [21,44,48], we observe that for the *Control* condition (top plot of Fig 3B) the initiation points are focused in a relatively small area, indicating strong similarity between all the network bursts. More varied burst initiation sites are observed in the *Tracks* (central plot) and the *Squares* (bottom) conditions. The former displays the broadest spatial distribution of initiation points, illustrating the rich repertoire of activity patterns in the *Tracks* condition, while the *Squares* condition falls in between the *Control* and *Tracks* conditions.

The strong localization of the front initiation site (Fig 3B–3C) agrees with previously published data and numerical studies [21,48]. The proposed mechanism is that metric correlations, inherited from the spatial embedding of neurons and connections, amplify random activations of the rest of the network in an avalanche-like manner towards basins of attraction (the initiation sites). Once an activity threshold in the site is crossed [49], a small group of neurons initiate a network burst that takes the form of a fast propagating activity front. The fast front propagation synchronizes the whole network, so that after a network burst the whole neuronal population is in a refractory state. Since the initiation site was the first to activate, it is likely that it will be the first site to recover from the strong activation, and hence is the most likely candidate to initiate the next network burst. In a sense the network becomes enslaved to a large extent to this initiation site.

Lastly, we consider the summary of front propagation velocities in the different conditions. Fig 3C shows that uninterrupted fronts (*Control* condition) propagate, on average, at $v_{C}=199\;\pm \;32\text{ mm}/\text{s}$, an order of magnitude faster than the obstructed fronts, with $v_{T}=21\;\pm \;12\text{ mm}/\text{s}$ and $v_{S}=69\;\pm \;25\text{ mm}/\text{s}$ for *Tracks* and *Squares*, respectively. The fronts in the *Squares* conditions propagate faster than in the *Tracks* one, highlighting again their intermediate role, albeit more similar to the *Tracks* condition. We note that, for *Tracks*, the measured velocities correspond to fronts propagating perpendicular to their orientation. The velocity within a single track is very fast and similar to the one observed in the *Control* case.

#### Impact of obstacle height *h* and internal noise intensity *σ* on activity propagation.

Here we considered only the data with the *Tracks* configuration, given the strong asymmetry the obstacles imposed in the directions parallel or perpendicular to tracks. For this data, and following the above analysis, the front velocity is effectually measured along the tracks’ transverse direction only.

In general, we observed an interesting dependence of the front propagation velocity on obstacle height *h* and internal noise intensity *σ*. As shown in Fig 3D, in the absence of obstacles (h=0 mm) the *Tracks* condition reduces to the *Control* one, and therefore the fronts propagate with high velocities, for the three noise amplitudes. However, a slight increase to h=0.1 mm results in an order of magnitude drop in front velocity for all noise amplitudes, which remains for h>0.1 mm.

Focusing on the effect of different noise amplitudes *σ*, Fig 3D shows that for h=0 mm a higher noise intensity leads to lower propagation velocities. This can be understood as wavefront break-ups, due to some of the neurons just ahead of the wavefront being in a refractory state due to spontaneous activation. In contrast, for increased obstacle height h≥0.1 mm we see that stronger noise leads to some fronts with increased velocities. We hypothesize that this can be explained using the concept of a firing “quorum” [49]: the idea that a neuron needs a minimal number of contemporaneously incoming excitations in order to be activated. Increased noise driving leads to an increase in neurons spontaneously activating. Given the low amount of connections crossing from one track to the next such an increase in spontaneous activation is beneficial, by leading to either a higher number of active neurons in the originating track (in order to utilize all the traversing connections), or to the activation of some of the neurons in the receiving track.

Lastly, we observe that increasing the noise driving *σ* leads to an increase in the total number of bursts, but that changing obstacle height *h* does not strongly affects the number of bursts (Fig 3E and S1 Appendix).

### Structural connectivity traits that shape network dynamics

It is clear from the previous sections that different growth conditions affect the activity dynamics of the resulting culture differently, and they do so through their influence on the network connectivity. A major advantage of numerical simulations over experiments is that one can access the structural network connectivity directly, a feature normally not accessible in experiments. In this section we look at the structural connectivity matrices resulting from the network growth algorithm for h=0.1 mm and ⟨ℓ⟩=1 mm, and graph-theoretical measures derived from these matrices.

**Fig 4:**
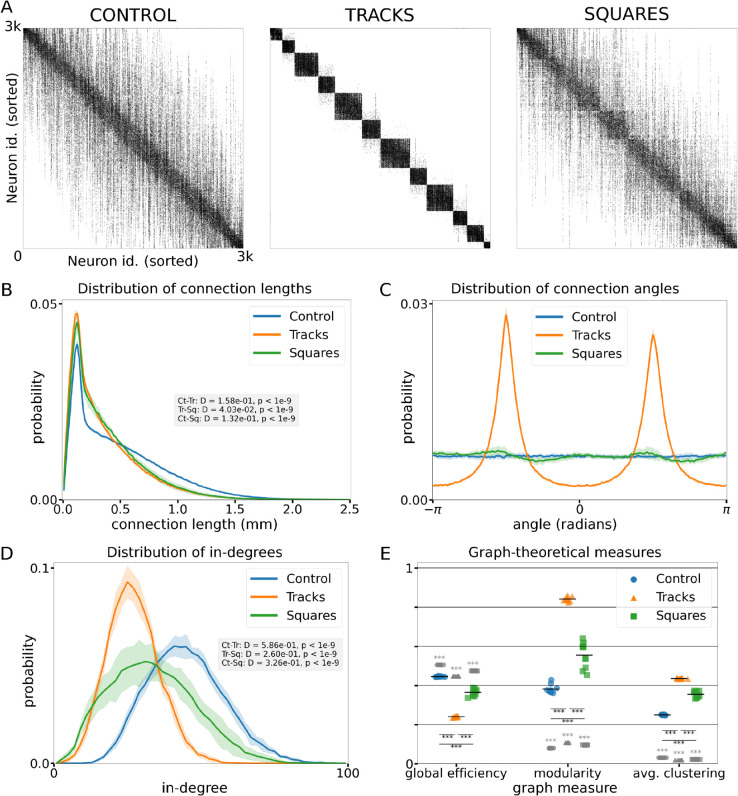
Structural connectivity of simulated neuronal culture growth. **(A)** Representative structural connectivity matrices for the three conditions. Black dots indicate that a connection exists between the neurons represented in the two axes. Neurons are ordered according to their position in the neuronal culture from left to right. **(B)** Distribution of connection lengths between neurons for the conditions. The gray inset shows two-sample Kolmogorov-Smirnov tests. **(C)** Distribution of angles between connected neurons. **(D)** Distribution of number of incoming connections. The gray box shows two-sample Kolmogorov-Smirnov tests. **(E)** Several graph-theoretical measures (from left to right: global efficiency, modularity *Q*, and average clustering) of the structural connectivity matrices (in color) and the values for random Erdös-Rény graphs with average in-degree matched to the average of each condition (in gray). Stars indicate significance of two-sample unpaired Student’s t-tests as follows: *: *p* < 0.05; **: *p* < 0.01; ***: *p* < 0.001. Significance indication below horizontal black lines indicate significance of tests across conditions. Significance indications above the gray markers indicate the significance of tests between the null distributions (indicated in gray) and the measured distributions (in color). Parameters used for all panels: h=0.1 mm and ⟨ℓ⟩=1 mm, panels B-E contain data of 10 independently grown networks per condition.

Illustrative structural adjacency matrices, with neurons sorted by their horizontal position, are shown in Fig 4A for each of the three conditions. As expected, the *Control* condition shows a distance-dependent connectivity profile, in which connections are most probable close to the diagonal, i.e. between nearby neurons, and are less probable off-diagonal, mirroring the distance dependent nature of the connection algorithm. In contrast, the *Tracks* condition shows a clear modular connectivity blueprint, with differently sized boxes along the diagonal indicating strongly connected modules of nearby neurons (within the same track), with few connections between neurons from neighboring tracks and nearly no connections extending beyond neighboring tracks. Lastly, the *Squares* condition displays again an intermediate state. Some modular structure is discernible as in the *Tracks* condition, yet largely the unmodulated distance-dependent connectivity profile of the *Control* condition is followed. These results show that what we can observe in the dynamics of the network, such as module-level, integrated versus segregated dynamics, can be linked clearly to the characteristics of the structural connectivity matrices.

Besides inspecting the structural connectivity matrices qualitatively, it is useful to look at the differences between the three conditions using coarse-grained observables. Fig 4B–4E show a number of network measures calculated from the structural connectivity matrices. Looking at the distribution of connection lengths (Fig 4B) it is apparent that there are predominantly short-range connections, of typically 0.20 mm, in all three conditions. The *Tracks* and *Squares* conditions exhibit very similar distributions, with a short tail vanishing at about 1.5 mm, showing that the presence of obstacles confines most of the connections to a relatively small space. In contrast, the *Control* condition shows a heavier and longer tail vanishing at 2 mm. The differences in the distribution of connection lengths conform to those of the neuronal dynamics in the different conditions, especially —and as expected— to the co-activation sizes (Fig 2D) and propagation velocities (Fig 3C).

Fig 4C shows the distribution of the angle between the projecting and receiving neuron for all connections for the different conditions. The *Tracks* condition shows that there is a clear predominance of connections at ±π/2 radians, which correspond to connections that remain confined to each track, whereas for the *Control* and *Squares* condition no such bias exists.

Given the differences in the distance that the axons traverse in the different conditions, as apparent from Fig 4A–4B, it is expected that different conditions also lead to different in-degree distributions, since axons confined to smaller spaces pass less unique neurons to connect to. Fig 4D shows the in-degree distributions for the three conditions. In accordance with the expectation, the *Tracks* condition shows a narrow distribution centered at a low number of connections. Both the *Control* and *Squares* conditions show distributions with similar shapes and broader than the *Tracks* condition, indicating a wider in-degree range, largely due to the presence of more high-degree nodes than in the *Tracks* condition. Moreover, the distribution of the *Control* condition is shifted towards higher in-degrees, owing to the free growth of the axons compared to the slight confinement in the *Squares* condition.

Lastly, Fig 4E shows three graph-theoretical measures calculated on the structural connectivity matrices. The first measure is the unweighted topological global efficiency, which is the inverse of the average path length between pairs of neurons. As expected, the global efficiency of the *Control* case is the largest, whereas the *Tracks* condition has the lowest global efficiency and the *Squares* is again intermediate. However, differences between the three conditions are small, which can be understood by the fact that all three have a strong bias towards short-range connections (Fig 4A–4B), and thus in all three conditions a neuron needs several steps on average to reach distant neurons. The second measure shown in this plot is the modularity *Q*, which quantifies the degree of modular structure in the network. The *Tracks* condition displays the most modular structure, characterized by the highest modularity *Q* value, followed by the *Squares* and lastly by the *Control* condition. A high *Q* for *Tracks* is expected, since conceptually neurons interconnect more strongly along tracks than across them, effectually shaping a system of interconnected modules that are the tracks themselves. Finally, the average clustering coefficient measures the number of closed triangles versus the total number of triangles in the network. More strongly clustered or modular networks are expected to have a higher average clustering coefficient, and as expected the *Tracks* condition has the highest value. However, both the *Squares* and *Control* conditions have very similar values, indicating again the predominance of local connectivity. To rationalize the impact of spatial constraints, we have compared the above network measures with null models based on random Erdös-Rény graphs that preserve the same average in-degree of each condition. The results (gray symbols) indicate that, in all spatial networks, the network measures are significantly higher than in the corresponding null models. This result is important since it evinces the strong capacity of spatial embedding to drive metric correlations and imprint non-random topological features, even in the relatively simple scenario of neurons growing on a flat two-dimensional substrate.

### Spatial anisotropy and external noise driving

The activity dynamics shown above for the *Tracks* condition shows that there are two (experimentally modifiable) parameters that drive the differences in the behavior of neuronal cultures: (i) the intensity *σ* of the noise driving, which influences the ease with which neurons activate, and (ii) the obstacle height *h*, which determines the strength of the anisotropy. The latter leads to an increase in modularity of the network that can be interpreted as a source of a spatially quenched disturbance. In this section, we first investigate more extensively the effect of these two parameters on the dynamics of the neuronal culture. In the next section we use those results to find a relationship between the dynamical regime of the neuronal culture and the accuracy of structural connectivity reconstruction from the neuronal activity, using generalised transfer entropy.

#### Dynamical richness under different levels of anisotropy and noise.

**Fig 5:**
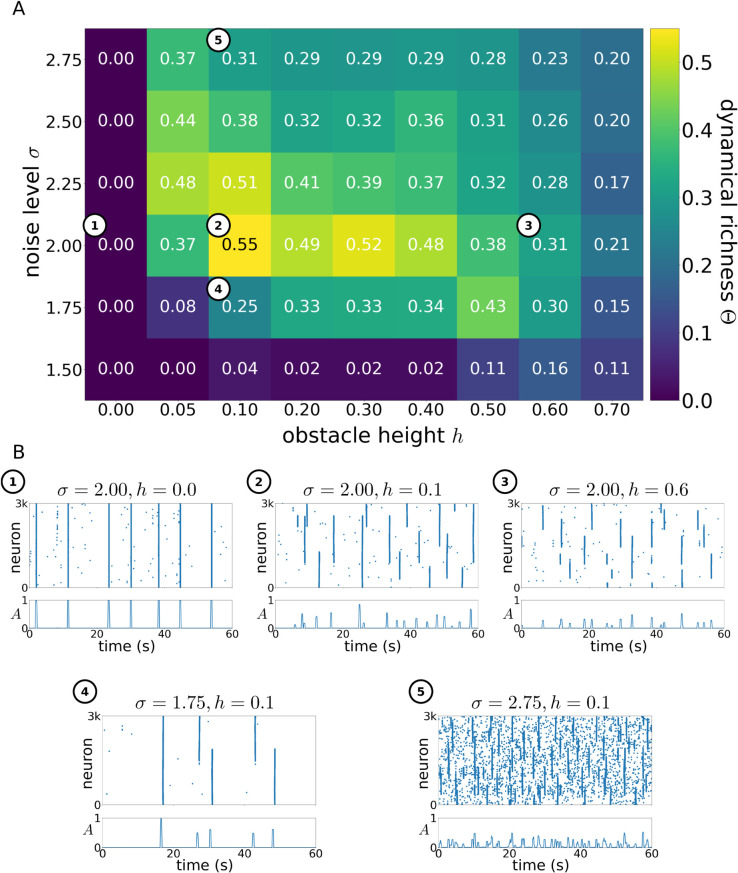
Effects of spatial anisotropy and external noise intensity on dynamics. **(A)** Dynamical richness Θ as a function of obstacle height *h* and noise intensity *σ* for the *Tracks* condition. Data shown is averaged over three reproductions. White-circled numbers relate to highlighted plots in panel (B). By definition Θ∈[0,1], but typically Θ⪅0.6. A value of Θ≳0.3 indicates dynamically rich activity. **(B)** Representative raster-plots of the simulation runs with parameters as marked in panel (A), illustrating the differences in activity dynamics for the different parameter values and measured dynamical richness. For all panels ⟨ℓ⟩=1 mm. Panel A shows averaged data of 3 independent simulation runs per parameter combination.

In order to understand the combined effect that the internal noise and the obstacle-driven anisotropy have on the dynamics, we calculate the dynamical richness Θ (see Methods) for different realizations of the neuronal culture as a function of the parameters *σ* and *h*. Fig 5A shows that intermediate values of both the noise intensity and the obstacle height *h* (such as situation 2 in Fig 5) lead to a dynamically richer activity with a maximal repertoire of co-activation sizes. In contrast, for extreme parameter values the activity is either predominantly synchronized (situations 1 and 4 in the figure), or dominated by small-scale activations (situations 3 and 5).

The noise intensity *σ* and obstacle height *h* lead to differences in the co-activation dynamics on different scales, mirroring the differences in scale that these parameters affect. Comparing the raster plots of situations 1 and 3, which correspond to no spatial anisotropy (h=0 mm) and a very strong anisotropy (h=0.6 mm), respectively, we see that the synchrony-breakup induced by the obstacles occurs on the scale of single tracks. In contrast, by comparing situations 4 and 5, corresponding to low (σ=1.75) and high (σ=2.75) noise intensities for the same obstacle height (h=0.1 mm), we observe that increased noise leads to the modules breaking up into randomly activating single neurons, with occasional synchronization of single modules.

Together, these results show that for both the external noise *σ* and the spatial anisotropy *h*, optimal intensities exist that maximise the dynamical repertoire of the neuronal culture. These observations are important, since they may inspire experimentalists to design more elaborate *in vitro* networks that mimic brain-like dynamics, i.e. activity that is neither fully synchronous nor random. Additionally, the ability to bring a neuronal network to a state with an activity that is intrinsically rich appears as a key ingredient for optimal processing of input stimuli.

### Reconstruction of structural connectivity under different noise conditions

The inference of structural connectivity between neurons from their recorded activity is still an open challenge, yet it is important for understanding the functioning of neuronal networks [50,51]. Transfer Entropy [52], and its extension for neuronal cultures called Generalised Transfer Entropy (GTE) [53,54], are extensively used tools to estimate the connectivity between neurons. However, synchronized activity can act as a confounding factor in the estimation of neuronal connection strengths using TE [50,53,54]. For instance, when the network behavior is strongly dominated by culture-wide synchronous activity, TE over-estimates the number of connections. In fact, GTE was proposed to deal with this by focusing the analysis on periods of non-synchronized activity [53]. However, when strongly synchronized activity dominates the culture, there might not be sufficient data available for a reliable analysis. In the previous section, it was shown that increased noise driving and spatial anisotropy lead to a desynchronization of the neuronal activations. Hence, a natural question arises whether GTE network inference works better for more modular *h* > 0 and noisier σ>2.00 *in silico* cultures.

**Fig 6:**
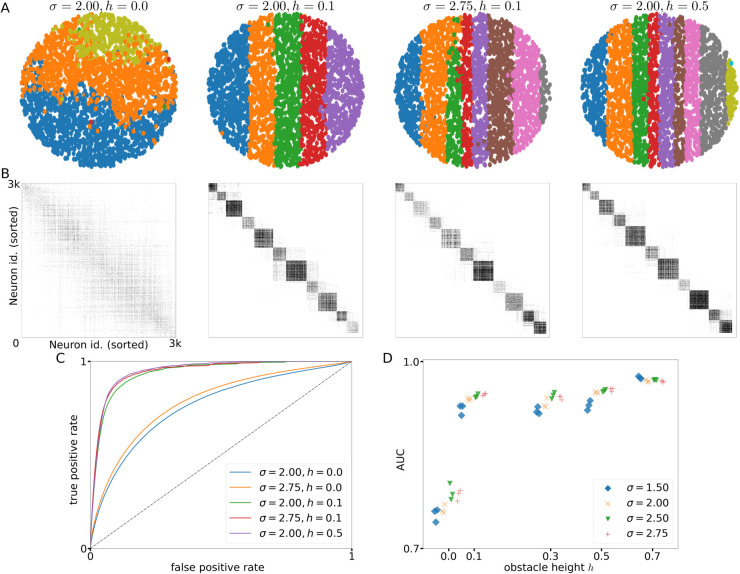
Effective connectivity analysis. **(A)** Representative network maps, color-coded to indicate the functional modules that the neurons belong to, as found by estimating the effective connections of neurons using GTE and the Louvain algorithm, for different parameter values (different columns) for the *Tracks* condition. **(B)** Effective connectivity matrices as given by thresholded GTE measures, corresponding to the networks in panel (A). Neurons are ordered as Fig 4A with the leftmost neuron at index 0 and the rightmost neuron at *N*. **(C)** Receiver Operating Characteristic (ROC) curves for quantifying the resemblance between structural and effective network connectivity, for different parameter values. **(D)** Area Under the Curve (AUC) values of the ROC curves for a range of obstacle heights *h* and noise amplitudes *σ*. For all panels ⟨ℓ⟩=1 mm.

To address this question, we calculated the GTE values for the simulated neuronal cultures with different noise amplitudes *σ* and obstacle heights *h*, keeping the condition *h* = 0 as reference corresponding to a control network with no spatial anisotropies. Both the spatial network maps (Fig 6A), in which neurons are grouped into functional modules using the Louvain algorithm [55], and the effective connectivity matrices (Fig 6B) show qualitative agreement with the structural network plots in Fig 2A and the structural connectivity matrices in Fig 4A.

In order to quantify the accuracy of the reconstruction of individual connections, we constructed Receiver Operating Characteristic (ROC) curves for the different parameter combinations. As Fig 6C shows, the reconstruction quality separates into two groups. For spatially isotropic cultures h=0 mm (blue and orange lines in Fig 6C) the GTE method has a fair performance, with an area under the curve (AUC) near 0.8 for all noise amplitudes, as shown in Fig 6D. The presence of even a slight spatial anisotropy h=0.1 mm increases the quality of the GTE reconstruction to an AUC value of 0.9, with a very small increase in performance for higher noise amplitudes *σ* (purple vs green line in Fig 6C). Further increase of the obstacle height only slightly increases the reconstruction performance (red vs green line in Fig 6C). Hence, primarily, the increased separation of the activity dynamics into modular tracks leads to an increased capacity to reconstruct the structural connectivity using GTE, more than promoting sparse activation through increased noise. This is possibly explained by the fact that effective connections between pairs of neurons can exist mediated by several intermediate neurons, leading to very long effective connections between neurons that are not connected directly. The confinement of activity to largely separated tracks limits the spatial extent of effective connections to the same track, while at the same time the higher modularity of the structural connections increases the probability that any two neurons within the same track are connected. In contrast, increasing the noise intensity decouples the activity of single neurons, which is an effect that can be both advantageous and detrimental to the reconstruction of structural connections from effective connections, since for noise to be advantageous the activity of neurons need to be decoupled from the global activity, yet smaller scale synchronization between connected neurons is needed for GTE to be able to measure their connection. Fig 6C shows that for slight anisotropy h=0.1 mm and a high noise intensity σ=2.75 the reconstruction is as good as for a very strong anisotropy h=0.5 mm and a intermediate noise intensity σ=2.00 (compare red and purple lines in the figure), indicating that the presence of both spatial anisotropy and noise driving helps to decouple the activity of the neurons from culture wide synchrony.

The observation that spatial anisotropies are an important factor in reconstruction accuracy is interesting. There has been abundant discussion in the literature on the capacity to precisely unravel the connectivity blueprint of a neuronal network from activity data only [54,56], since the intrinsic nonlinear nature of neurons in combination with intrinsic noise and variability of neuronal types makes the *inverse problem* of perfect reconstruction unattainable, requiring the incorporation of additional tools such as interrogation or labeling of neurons and connections. Our study shows that even a small restriction or guidance of neuronal connectivity by topographical anisotropy suffices to largely restrict the available connectivity repertoire and substantially improve reconstruction performance.

## Discussion

The use of computational models has a long history within (computational) neuroscience, and has been valuable for the prediction, validation and interpretation of experimental results, as well as for the exploration of aspects that are inaccessible in biological experiments. Inspired by recent experimental results [11,12,44] and ongoing interest in growing neuronal cultures in structured environments, in this paper we have presented a numerical model of the growth and activity of *in vitro* neuronal cultures in the presence of obstacles in the growth environment. The activity of the simulated neuronal cultures agree qualitatively well with previously published experimental data [44], capturing the qualitative differences between the different growth environments for the overall dynamics (Fig 2B, 2C and 2E), the sizes of network co-activations (Fig 2D) and the spatio-temporal characteristics of collective activity (Fig 3A–3C). Moreover, the numerical model allows to investigate the effect of the inhomogeneous growth environment on the structural network connections (Fig 4) and the influence of the magnitude of structural anisotropy and noise on the activity (Fig 5) and reconstruction of structural connections from activity data (Fig 6).

There is a quantitative difference between the activity of the numerical model and the biological data. Prominently, the rate of collective network bursts is slower for experimental (Fig 2B and 2C, left most panels) than the numerical (Figs 2B and 2C, 3th-column) data. A more careful tuning of parameters could result in a closer match between numerical and biological bursting rates. However, our present aim was to qualitatively capture the differences in network activity under the different growth environments, rather than exactly match the experiment statistics. For instance, we have carried out simulations with a range of values for synaptic depression $\tau_{q}$, which controls the characteristic inter-burst interval, and found that the differences observed across conditions hold. Moreover, the differences in bursting rate between experiments and model could also be explained by the differences in system size, and the activity of homeostatic plasticity in biological neuronal cultures.

Additionally, we have simulated around 2,800 neurons per culture, whereas the experimental data contain on the order of 10^4^ neurons. It is possible that larger networks take more time for activity to quiet down following a network burst, and hence deplete the synaptic vesicle reservoir of each neuron more strongly during a burst, leading to an increased time between bursts.

In the interest of simplicity, we have only included the minimal set of required dynamical variables to capture qualitatively the activity of neuronal cultures. Yet, biological neuronal networks have a plethora of synaptic plasticity and homeostatic regulatory mechanisms that keep the firing and bursting rates within certain bounds [57]. Scaling of the total of incoming connections to each neuron, adjusting the neuronal excitability, or the magnitude of spontaneous activity, all can adjust the rate of network bursting, and are all present in biological neuronal cultures. A future direction would therefore be to include these factors in the presented model.

### Development of network structure

A crucial part of the presented model is the network growth algorithm. In general it follows an established idea: that connections can only be formed when a part of the pre-synaptic neuron (often the axon) is in close proximity to a part of the post-synaptic neuron (often a dendrite) [58–60].

Our model is based on the work of Orlandi *et al.* [48], in which the axon growth was modeled for homogeneous growth environments (*Control* cultures in our work), based on experimental observations [9,61,62]. Importantly, the network growth algorithm results in a network that is a geometric random graph (in which connection probability decreases with distance between neurons), yet with the projections of a single neuron having strongly correlated directions due to the axon growth. The growth algorithm can be made more biologically plausible by including the growing of dendrites, the branching of neurites (axons and dendrites), and the inclusion of different growth characteristics for different types of neurons [63]. This would result in more biologically realistic connectivity, and it is interesting to investigate the different activity dynamics that will result. On the other hand, for the sake of simplicity, an often used technique is to consider a distance-dependent connection probability [21,64]. This leads to geometric random graphs which have the advantage of being fast to implement and to be mathematically tractable up to a certain extent. The inclusion of long range correlated “patchy” connectivity [64] gives the possibility to include more realistic (cortical) wiring [65]. These correlated connections can emerge through sensory input through plasticity mechanisms, akin to the formation of receptive fields. Interestingly, patchy connectivity can also be obtained with the algorithm presented in the current paper, by including the tendency of axons to follow other axons and in this way form axon bundles, which would lead to correlated projections of nearby neurons.

In the model presented here, the number of synapses increases monotonically with increasing axon length, leading to a monotonic increase in the average number of incoming connections (S1 FigA and S2 FigA). This is in agreement with *in vitro* observations [27], where the average number of synapses per neuron increases. However, in contrast to the referenced paper where the increase in synapses per neuron occurs as a combination between a reduction in the total number of synapses and a contemporaneous decrease in the number of neurons, in our case the number of neurons stays stable across development. One can interpret the case presented by us as only tracking those neurons that survive until the latest developmental stages, and thus those neurons that have an overall increase in synapses per neuron along development. We have, for the sake of simplicity, not included any synaptic plasticity or adaptation mechanisms which are indubitably at play in biological networks. Neither have we considered any neuronal apoptosis.

The effect of increased number of connections, arising either through an increase in axon length or by an increased density of neurons, has—expectedly—an effect on the activity of the network. S2 FigB shows how the co-activation sizes change for different combinations of average axon length ⟨ℓ⟩ and neuron density *ρ*, keeping all other parameters fixed, for the *Tracks* condition (with h=0.1 mm). For increased number of connections (marked with III and V in S2 Fig), the average co-activation size increases, whereas lower number of connections (marked with II and IV) result in lower sizes of co-activation.

### Role of obstacle density

Around 25% of the surface is covered by PDMS squares in the *Squares* condition used in the experimental data [44] and the numerical results presented here. In contrast, for the *Tracks* condition 40% of the environment is covered in PDMS. Hence, the question arises whether the differences found between the *Tracks* and *Squares* conditions are due to the differences in area covered in PDMS, or some difference imposed by the type of anisotropy. S3 Fig shows comparisons of some measures on the structural connectivity of *Tracks*, *Squares* covering 25% of the substrate (*Sq.* 25%) and *Squares* covering 40% of the surface (*Sq.* 40%). Visual inspection shows that the difference between covering 25% or 40% of the surface with PDMS squares is smaller than between both *Squares* conditions and the *Tracks* condition with respect to the in-degree distribution (S3 FigA) or connectivity lengths (S3 FigB). Kolmogorov-Smirnov tests find significant differences between all three distributions for both measures. The graph measures interestingly places the 40% *Squares* condition between the 25% *Squares* and the *Tracks* networks (S3 FigC), indicating that the density accounts for some of the differences found between the 25% *Squares* and *Tracks*, yet cannot explain their differences fully.

The influence of the PDMS obstacle density on the network activity has not been explored further in this paper, but is an interesting question. Currently *in vitro* and numerical experiments are being carried out to explore this question for a future publication.

### Excitation and inhibition

In our study we considered both excitatory and inhibitory neurons, to mimic existing experimental designs [44]. The impact of the excitatory-inhibitory balance was not investigated in depth in the present work. However, we carried out exploratory simulations in which inhibitory neurons were inactivated, observing that network dynamics evolved towards stronger bursting. This result is in agreement with diverse experimental and numerical studies [12,48,66] describing that blocked inhibition reduces the capacity of the network to accommodate rich and diverse dynamical states, driving the network towards a pathological-like hyper-synchronous state. Since the balance between excitation and inhibition is altered in neurological diseases such as epilepsy and genetic forms of Parkinson’s, which have been investigated *in vitro* [67,68], our work may help experimentalists to engineer neuronal cultures that achieve a rich repertoire of activity patterns in healthy conditions to then explore the degradation in dynamics as inhibition is gradually lost.

### Reconstruction of connectivity

Finally, we observe that GTE-reconstructed connectivity approaches well the underpinned structural network in the case of anisotropic cultures. This evinces that imprinted spatial constraints favor the occurrence of overt neuron-to-neuron interactions which, as a counterbalance to network-wide bursting or random activity, is the key ingredient to bring to light the key topological and organizational traits of the structural connectivity. Our results fit well with the experimental work of Montalà-Flaquer *et al.* [44], in which the *Tracks* configuration was seen to exhibit an abundance of effective connections oriented along the tracks themselves, a feature that was independently confirmed through immunostaining. Thus, our study provides a strong *in silico* evidence to support the development of neuroengineered neuronal cultures, i.e., those systems where neurons and connections are tailored to fit *ad hoc* configurations and activity patterns. These configurations not only imprint rich dynamical traits that resemble brain dynamics, but their topological features can be predicted and analyzed. This may be crucial to understand the processing of input stimuli in a neuronal network, where a wealth of ingredients play an important yet elusive role, including network architecture, noise, and the balance between excitation and inhibition [69].

## Conclusions

In this work we constructed an *in silico* model to replicate the connectivity structure and dynamics of laboratory-grown neuronal cultures characterized by spatial anisotropies, which mold and dictate the layout of connections in the network [44]. The imprinted anisotropies, leading to non-uniform connectivity probabilities among neurons in spatially-confined regions, led to activity patterns that substantially departed from the control scenario of free connectivity. The simulated networks not only reproduced the experimental behavior, but also provided valuable insight on the role of the two key ingredients that govern collective behavior, namely the strength of the spatial anisotropy and the noise intensity that drives spontaneous activity. The results show that, whereas the dynamics in control (non-anisotropic) networks is dominated by regular bursting episodes that encompass the entire system, the presence of inhomogeneities markedly favors a much richer repertoire of activity characterized by a broad range of co-activation patterns. However, both the spatial anisotropy strength and noise intensity have to be mild in order to maximize the richness of collective activity, otherwise the system is locked into extreme states of permanent bursting or random driving of activity in spatially isolated areas. Thus, our work is inspirational for those studies, both *in silico* and *in vitro*, that aim at understanding, and even mimicking, the richness of brain-like dynamics and its relation to the network’s building blocks, neurons and connections.

## Materials and methods

Numerical simulations aimed at replicating the two-level topographical patterns of the experiments described by Montalà-Flaquer *et al.* [44] in which, as sketched in Fig 1A, a PDMS mold contained vertical modulations in the form of parallel tracks with a height *h*. Neurons (80% excitatory and 20% inhibitory) preferentially connected along the tracks either at the bottom or at the top of the mold. Neurons at the bottom could project connections to neurons at the top with some probability $P_{\text{bottom}\to \text{top}}$, and neurons from the top to the bottom with probability $P_{\text{top}\to \text{bottom}}$, effectually shaping a globally interlinked system yet with most connections along the tracks.

To realize such a scenario *in silico*, neuronal networks were modeled in two-dimensional circular cultures with radius r=1.5 mm, on which neurons were plated with a uniform density of $\rho =400\text{ neurons}/{\text{mm}}^{2}$, resulting in $N=\lfloor \rho \pi r^{2}\rfloor \approx 2800$ neurons in total (Fig 1B). The *N* neurons of the network were placed randomly on a circular area in such a way that somas (circular areas of radius $r_{soma}=7.5\;\mu \text{m}$) of neighboring neurons did not overlap. The network connections were determined following the algorithm of Orlandi *et al.* [48], complemented with the influence of the PDMS topography on axon growth, as described below.

The code necessary to run the numerical simulations is available at https://doi.org/10.5281/zenodo.17256850 and https://github.com/akkeh/nc_sim.git.

### Growing the networks

Starting from the center of each neuron *i*, axon growth was modeled by concatenating line segments of length Δℓ=10 μm up to a total length $\ell_{i}$, which for each neuron was independently drawn from a Rayleigh distribution with mean $\mu_{\ell }$. Each consecutive segment was placed at an angle drawn from a Gaussian distribution with zero mean and standard deviation $\sigma_{\phi }=0.1\text{ radians}$ with respect to the direction of the previous segment, as illustrated in Fig 1B. The dendritic tree of each neuron *i* was modeled as a circular area of radius $r_{\text{dr}}^{\left(i\right)}$, drawn from a Gaussian distribution with mean $\mu_{\text{dr}}=150\;\mu \text{m}$ and standard deviation $\sigma_{\text{dr}}=20\;\mu \text{m}$.

During the growth of the axons, whenever a line segment was placed such that it crossed the border of an obstacle, two things could happen depending on (i) the angle between the axon and the border, and (ii) the height of the obstacle. On the one hand, if the angle between the axon line segment and the border was smaller than $30^{\circ }$, the segment was replaced by one parallel to the border plus a random deviation on the order of $\sigma_{\phi }$, as illustrated in the detailed map of Fig 1B (orange and green axons). On the other hand, if the angle between the line segment and the border was larger than $30^{\circ }$, the segment remained there with probability *P*(*h*,*d*), to simulate the crossing from a top to bottom obstacle or vice versa (Fig 1B, blue axon). The probability *P*(*h*,*d*) depended on the height *h* of the obstacle and the direction d∈{bottom→top, top→bottom} that the axon followed when crossing the obstacle border. If this probability *P*(*h*,*d*) was satisfied, the axon crossed the obstacle border and the line segment was counted to have a length of Δℓ+h μm. With complementary probability 1–*P*(*h*,*d*) the axon was again deflected, meaning that it was replaced by a segment parallel to the border. The numerical values used to quantify these probabilities of axonal crossing for different heights were obtained from experimental data [47] as explained below, and are provided in Table 1.

Once all axons were placed on the substrate, a connection between neurons *i* and *j* was established whenever the axon of neuron *i* crossed the dendritic tree of neuron *j* with a probability α=0.5. The set of network connections was stored in the *structural* adjacency matrix $S=\{s_{ij}\}$, with *s*_*ij*_ = 1 for the presence of a connection j→i and *s*_*ij*_ = 0 otherwise (Fig 1C). The weighted connectivity matrix $𝐖=\{w_{ij}\}$ is obtained from the structural matrix **S** by drawing a connection weight from a uniform distribution for each existing connection, $s_{ij}=1⟹w_{ij}∼U(0,1)$. The sign of the outgoing signal is determined by whether a neuron is excitatory (80% of the network) or inhibitory (remaining 20%), through the synaptic dynamics described in the next section.

### Neuronal dynamics

The dynamics of each neuron was governed by the Izhikevich two-dimensional quadratic integrate-and-fire model with adaptation [70], which provides a good balance between computational efficiency and biological accuracy, and was previously used for numerically simulating neuronal cultures [48,71]. The model is given by

$$\frac{dv_{i}}{dt}=0.04v_{i}^{2}+5v_{i}+140-u_{i}+\sum_{j=0}^{N}w_{ij}p_{j}+\sigma \eta_{i},\;\frac{du_{i}}{dt}=\epsilon (\rho v_{i}-u_{i}),$$ (1)

where $𝐖=\{w_{ij}\}$ is the above-defined adjacency matrix of connections (directed and weighted) between neurons, and $\eta_{i}$ is a Gaussian white noise term that influences the sporadic activation of each neuron *i*. The parameter *σ* quantifies the intensity of the white noise, and can be tuned to emulate the abundant spontaneous activity observed in biological neuronal networks.

The membrane potential $v_{i}$ and recovery variable *u*_*i*_ are reset once the membrane potential exceeds a threshold $v_{c}$ and the neuron is said to “spike”:

$$v_{i}(t)>v_{c}⟹\left\{ \begin{array}{cc} v_{i} & \leftarrow v_{0}\\ u_{i} & \leftarrow u_{i}+\delta_{u}. \end{array}\right.$$ (2)

After these resets, the dynamics is again governed by Eq (1).

Once a neuron elicits a spike, it sends signals to the neurons it is connected to. This signal is determined by the synaptic dynamics, governed by two variables, namely the synaptic potential *p*_*i*_ and the synaptic recovery variable *q*_*i*_. The synaptic potential *p*_*i*_ follows the equations

$$\tau_{p}^{\left(i\right)}\frac{dp_{i}}{dt}=-p_{i},\;v_{i}(t)>v_{c}⟹p_{i}\leftarrow p_{i}+\delta_{p}^{\left(i\right)}q_{i},$$ (3)

which describe the decay and release of neurotransmitters in the synaptic cleft, respectively, assuming the rising-phase of the neurotransmitter release to be instantaneous [72]. The decay time $\tau_{p}^{\left(i\right)}$ and reset constant $\delta_{p}^{\left(i\right)}$ depend on the type of neuron *i* (excitatory or inhibitory), specifically the reset constant $\delta_{p}^{\left(i\right)}$ will be non-negative (non-positive) for excitatory (inhibitory) neurons.

The synaptic recovery variable *q*_*i*_ accounts for the phenomenon of synaptic depression, according to which neurons that are tonically active experience a reduction in the efficacy of synaptic transmission [18,62]. This phenomenon can be described by an evolution equation and reset rule given by

$$\tau_{q}\frac{dq_{i}}{dt}=1-q_{i},\;v_{i}(t)>v_{c}⟹q_{i}\leftarrow (1-\delta_{q})q_{i}.$$ (4)

The parameters used in the numerical simulations are listed in Table 2.

### Determination of the obstacle-crossing probabilities

The probability of an axon crossing an obstacle wall is determined by using calcium imaging data of neuronal activity crossing a PDMS-glass border [47]. The axon crossing probabilities are then fitted by comparing the numerically simulated activity to this data.

The data consists of recordings of different neuronal cultures, each 6 mm in diameter, prepared on glass but with half of the culture covered with a PDMS layer of height *h*. The set of prepared cultures had different heights between the glass and the PDMS. This allowed to calculate the number of bursts that are initiated either in the glass part or the PDMS part of the plate, and to register the frequency at which the activity originating in either half propagates successfully across the glass-PDMS border. The second and third column of Table 1 show the crossing probabilities of the activity obtained for different PDMS heights.

Subsequently, numerical simulations are run with different values for axons crossing from glass to PDMS $P_{\text{glass}\to \text{PDMS}}$ and PDMS to glass $P_{\text{PDMS}\to \text{glass}}$, and the activity propagation across the glass-PDMS border are registered and compared with the experimental values. In case the fraction of activity crossing in a certain direction is too low (high) the corresponding axon crossing probability is increased (decreased) until the simulated activity propagation fraction agrees with the experimental ones within 5% precision. The obtained axon crossing probabilities are given in the last two columns of Table 1.

### Measures of neuronal activity

#### Population activity.

The population activity PA(t) indicates the number of active neurons within a short time window $\tau_{\text{win}}$ centered around time *t*, divided by the total number of neurons:


$$\text{PA}(t)=\frac{1}{N}\left|\left\{i\;:\;\exists_{t_{i}^{\left(k\right)}}|t_{i}^{\left(k\right)}-t|<\frac{\tau_{\text{win}}}{2}\right\}\right|.$$

In words, the population activity PA at time *t* is defined as the size of the set of neurons *i* for which there exists a *k*-th spike occurring at a time $t_{i}^{\left(k\right)}$ that is within a $\tau_{win}=200\text{ ms}$ time-window centered around the current time *t*. Sharp peaks in PA(t) reveal strong network co-activations, i.e. groups of neurons (from small ensembles to network bursts) that spike coherently within a short time window.

#### Dynamical richness.

Dynamical richness [11,73] Θ is a measure of the diversity of co-activation sizes. In the present work, Θ is calculated by creating a *m*-bin histogram of the peaks $\Gamma_{i}$ of the population activity PA(t) trace of a recording, resulting in an estimated distribution of peaks p(Γ), and following calculating the deviation of p(Γ) from a uniform distribution.


$$\Theta =1-\frac{m}{2(m-1)}\sum_{i=0}^{m-1}\left|p\left(\frac{i}{m}\leq \Gamma <\frac{i+1}{m}\right)-\frac{1}{m}\right|.$$

The quantity Θ attains values in the range [0,1]. Values of Θ close to 0 indicate that the network operates at full extremes of either random neuronal activity or fully synchronous network bursts, while values of Θ close to 1 indicate that all possible co-activation sizes are present in the system.

### Network measures

#### Effective connectivity.

Causal interactions between pairs of active neurons were computed through a GTE implementation [53] run in Matlab [74]. Specifically, pairs of neuronal activity trains *I* and *J* were constructed as binary series 600 s long with values of either ‘1’ (presence of activity) or ‘0’ (absence of activity), binned at 10 ms. For computation, and following Ref. [53], the Markov order was set to 2 and instant feedback was present. An effective connection between any pairs of neurons *I* and *J* in the network was then considered whenever the information contained in *I* significantly increased the capacity to predict future states of *J*. For that, raw transfer entropy estimates ${\text{TE}}_{I\to J}$ were first obtained, and then compared with the joint distribution of all inputs *X* to *J* and all outputs *I* to *Y* (for any X and Y), as

$$z_{I\to J}=\frac{{\text{TE}}_{I\to J}-\langle {\text{TE}}_{joint}\rangle }{\sigma_{\text{joint}}}.$$ (5)

Here $\left\langle {\text{TE}}_{joint}\right\rangle$ and $\sigma_{\text{joint}}$ are the average value of the joint distribution and its standard deviation, respectively. A significance threshold $z_{\text{th}}=2$ was established to accept an effective interaction as significant, setting $z_{I\to J}=1\;\forall \;z_{I\to J}\geq z_{\text{th}}$ and 0 otherwise. A threshold $z_{\text{th}}=2$ was set to compute effective connectivity data in the same way as the reference experimental work of Montalà-Flaquer *et al.* [44]. The derived effective connectivity matrices $E=\{e_{IJ}\}$ were therefore directed but binary, which allowed for a direct comparison with the underlying structural connectivity of the studied synthetic networks.

Throughout the text in the present work, the term ‘effective’ is used to refer to TE-inferred connections among neurons and the derived connectivity matrices. The term ‘functional’ is used to refer to the broader concept of network organization and characteristics.

#### Modularity analysis.

We used the modularity statistic *Q* to quantify the tendency of neurons to organize into groups (termed modules) that were strongly connected within themselves and sparsely connected with other groups [75]. This quantity is defined as:

$$Q=\frac{1}{2m}\sum_{i,j}\left(e_{ij}-\frac{k_{i}k_{j}}{2m}\right)\delta (c_{i},c_{j}),\;$$ (6)

where *N* is the number of neurons, *e*_*ij*_ represents the effective connectivity matrix, $k_{i}=\sum_{j=1}^{N}e_{ij}$ is the sum of the connections attached to neuron *i*, *c*_*i*_ is the community to which neuron *i* belongs, $m=(1/2)\sum_{i,j=1}^{N}e_{ij}$, and δ(u,v) is the Kronecker Delta with δ(u,v)=1 for u=v and 0 otherwise. *Q* varied between 0 (the entire network is the only module) and 1 (each neuron is a module), with intermediate values indicating the presence of modules of varying size. The optimal modular structure was computed using the Louvain algorithm [55]. In addition we have validated the results involving the Louvain algorithm using the Leiden algorithm [76] in order to rule out any spurious modularity imposed by the Louvain algorithm. The results were qualitatively the same. Detected modules were color-coded in the network maps to investigate whether modularity was related to the structure of the spatial disorder.

### Simulation parameters

The parameters used for the simulations are listed in Table 2. Simulations were run for typically 600 s to obtain sufficient co-activation events for statistics. For each condition (*Control*, *Tracks* or *Squares*), 10 network realizations were considered, except for the results in Fig 5, in which 3 repetitions per parameter-pair were considered.

## Supporting information

S1 FigAverage in-degree and connection distance along development.**(A)** Average in-degree $\left\langle k_{in}\right\rangle$ for different average axon lengths ⟨ℓ⟩ per condition. **(B)** Average connection length for different average axon lengths ⟨ℓ⟩ per condition.(PDF)

S2 FigRelationship between neuron density and average axon length, and average in-degrees in *Tracks* condition.**(A)** Average in-degree $\left\langle k_{in}\right\rangle$ as function of average axon length ⟨ℓ⟩ and neuron density *ρ*. **(B)** Distribution of co-activation sizes for five selected combinations of average axon length and neuron density, corresponding to parameters indicated in panel (A). Stars indicate significance of Mann-Whitney-Wilcoxon tests as follows: *: *p* < 0.05; **: *p* < 0.01; ***: *p* < 0.001.(PDF)

S3 Fig Structural connectivity measures for *Tracks*, *Squares* on 25% of the culture area, and *Squares* on 40% of the culture area (matched to the area occupied by *Tracks.* **(A)** Distribution of connection lengths between neurons. **(B)** Distribution of the in-degree. **(C)** Several graph-theoretical measures. For panels (A) and (B) the gray box reports results of two-sample Kolmogorov-Smirnov tests. The stars in panel (C) indicate significance of two-sample unpaired Student’s t-tests as follows: n.s.: *: *p* < 0.05; **: *p* < 0.01; ***: *p* < 0.001.
(PDF)

S1 Table Model description table. Following Nordlie et al. (2009) [77] we present the numerical model in table format.
(PDF)

S1 Appendix In the tables in this appendix the full results of the statistical tests carried out and indicated in the figures are provided.
(PDF)
