# Supplementary figures and images for: Role of connectivity anisotropies in the dynamics of cultured neuronal networks
Source: PLoS Comput Biol. 2025 Nov 6;21(11):e1012727. doi: 10.1371/journal.pcbi.1012727 (PMC12614803; doi:10.1371/journal.pcbi.1012727)

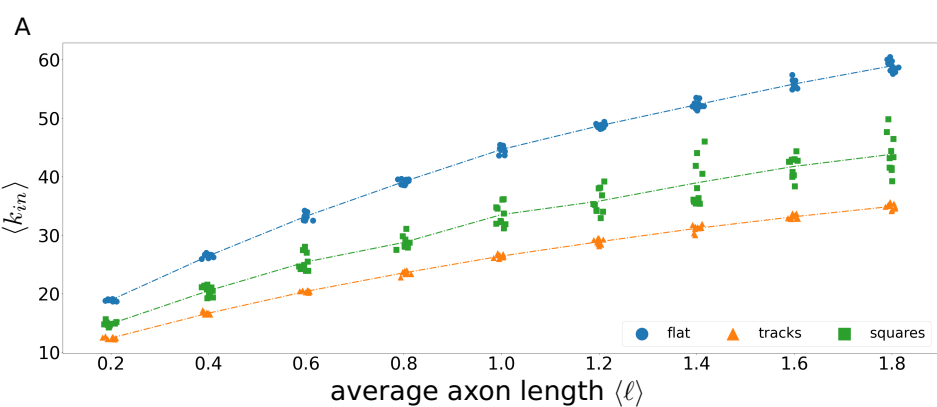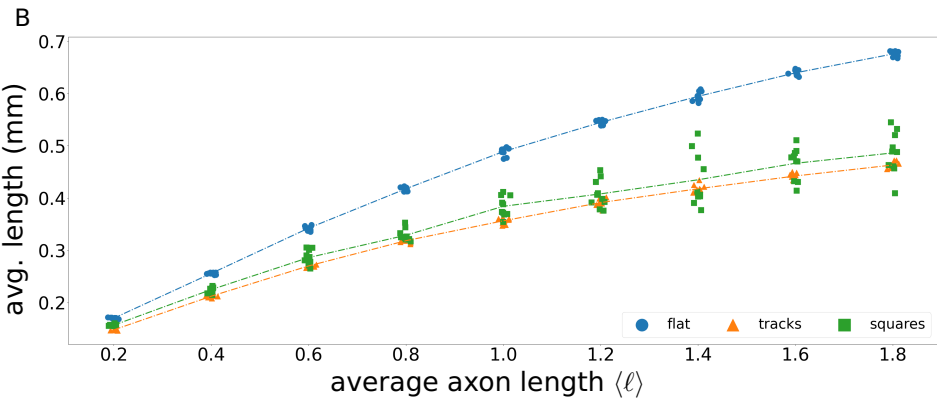

Supplement: S1 Fig — (A) Average in-degree ⟨kin⟩ for different average axon lengths ⟨ℓ⟩ per condition. (B) Average connection length for different average axon lengths ⟨ℓ⟩ per condition. (PDF) [file pcbi.1012727.s001.pdf]

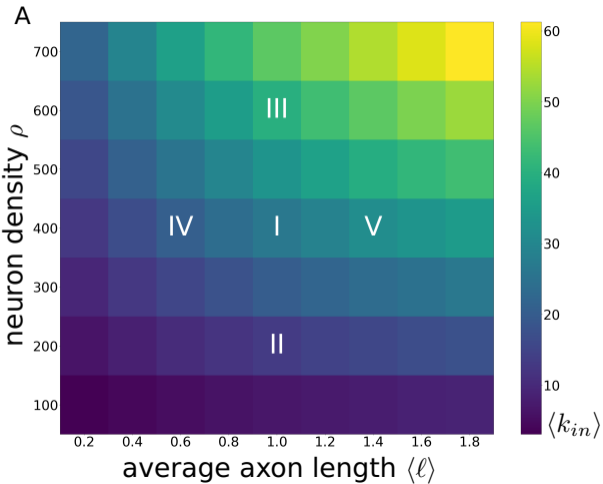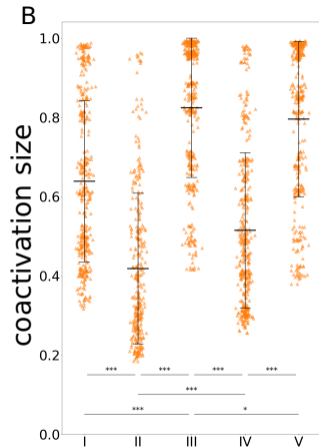

Supplement: S2 Fig — (A) Average in-degree ⟨kin⟩ as function of average axon length ⟨ℓ⟩ and neuron density ρ. (B) Distribution of co-activation sizes for five selected combinations of average axon length and neuron density, corresponding to parameters indicated in panel (A). Stars indicate significance of Mann-Whitney-Wilcoxon tests as follows: *: p < 0.05; **: p < 0.01; ***: p < 0.001. (PDF) [file pcbi.1012727.s002.pdf]

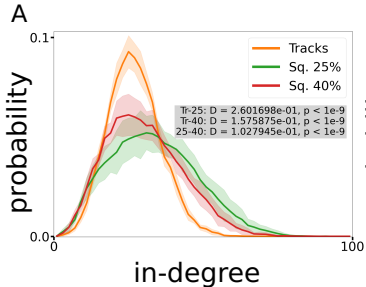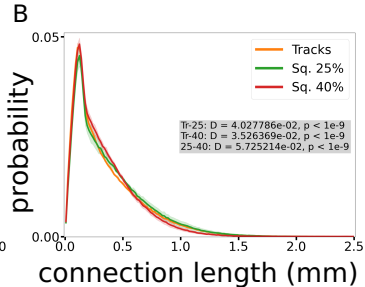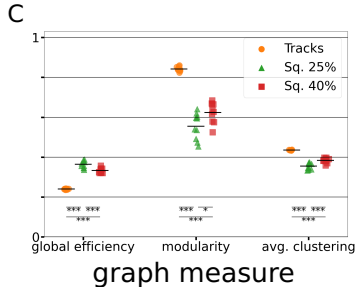

Supplement: S3 Fig — (A) Distribution of connection lengths between neurons. (B) Distribution of the in-degree. (C) Several graph-theoretical measures. For panels (A) and (B) the gray box reports results of two-sample Kolmogorov-Smirnov tests. The stars in panel (C) indicate significance of two-sample unpaired Student’s t-tests as follows: n.s.: *: p < 0.05; **: p < 0.01; ***: p < 0.001. (PDF) [file pcbi.1012727.s003.pdf]
