# Supplementary material for: Role of connectivity anisotropies in the dynamics of cultured neuronal networks
Source: PLoS Comput Biol. 2025 Nov 6;21(11):e1012727. doi: 10.1371/journal.pcbi.1012727 (PMC12614803; doi:10.1371/journal.pcbi.1012727)
Supplement: S1 Table — Following Nordlie et al. (2009) [77] we present the numerical model in table format. (PDF) [file pcbi.1012727.s004.pdf]

A	Model summary
**Populations**	Two: excitatory, inhibitory
**Topology**	Uniform distribution on two-dimensional disk
**Connectivity**	Resulting from growth algorithm (described in methods)
**Neuron model**	Izhikevich quadratic integrate-and-fire neurons with linear adaptation
**Channel models**	–
**Synapse model**	decaying exponential, short-time synaptic depression
**Plasticity**	–
**Input**	Gaussian white-noise
**Measurements**	Spiking activity

B	Populations	
**Name**	**Elements**	**Size**
E	Izhikevich simple neurons	$N_E = 0.8N$
I	Izhikevich simple neurons	$N_I = (1 - 0.8)N$

C	Connectivity		
**Name**	**Source**	**Target**	**Pattern**
E_{all}	E	E ∪ I	Following growth algorithm, weight $g_A \times U(0,1)$
I_{all}	I	E ∪ I	Following growth algorithm, weight $g_G \times U(0,1)$

D	Neuron and synapse model
**Type**	Izhikevich quadratic integrate-and-fire neurons with linear adaptation
**Subthreshold dynamcis**	$\frac{dv_i}{dt} = 0.04v_i^2 + 5v_i + 140 - u_i + \sum_{j=0}^{N} w_{ij}p_j + \sigma\eta_i,$ $\frac{du_i}{dt} = \epsilon(\rho v_i - u_i),$
**Spiking**	If $v_i(t) > v_th$ 1. $v_i \leftarrow v_0$ 2. $u_i \leftarrow u_i + \delta_u$ 3. $p_i \leftarrow p_i + \delta_p^{(i)} q_i$ 4. $q_i \leftarrow (1 - \delta_q)q_i$ 5. emit spike with timestamp t
**Synapse dynamics**	$\tau_p^{(i)} \frac{dp_i}{dt} = -p_i, \quad \tau_q \frac{dq_i}{dt} = 1 - q_i$

E	Input
**Name**	**Description**
Gaussian white noise η_i	Unique to each neuron i, $\langle \eta_i(t), \eta_j(t+\tau) \rangle = \delta(i-j)\delta(\tau)$

F	Measurements
Spike activity as raster plots	
