# Supplementary material for: Role of connectivity anisotropies in the dynamics of cultured neuronal networks
Source: PLoS Comput Biol. 2025 Nov 6;21(11):e1012727. doi: 10.1371/journal.pcbi.1012727 (PMC12614803; doi:10.1371/journal.pcbi.1012727)
Supplement: S1 Appendix — In the tables in this appendix the full results of the statistical tests carried out and indicated in the figures are provided. (PDF) [file pcbi.1012727.s005.pdf]

Table 1. Statistics for Fig. 2D. Statistics for the two-sample Mann-Whitney-Wilcoxon tests indicated in Fig. 2D.

**Numerical**				
$\langle \ell \rangle$	**comparison**	U **statistic**	p**-value**	N–N
0.20	Control – Tracks	1.79e+04	6.39e-44	132 – 137
0.20	Control – Squares	1.75e+04	3.78e-41	132 – 136
0.20	Tracks – Squares	7.43e+02	1.87e-39	137 – 136
0.40	Control – Tracks	2.08e+04	1.58e-47	139 – 151
0.40	Control – Squares	2.20e+04	2.44e-48	139 – 160
0.40	Tracks – Squares	6.61e+02	4.67e-47	151 – 160
0.60	Control – Tracks	2.30e+04	1.90e-50	147 – 157
0.60	Control – Squares	2.60e+04	4.17e-53	147 – 178
0.60	Tracks – Squares	1.76e+03	2.54e-43	157 – 178
0.80	Control – Tracks	2.03e+04	4.53e-48	143 – 142
0.80	Control – Squares	2.56e+04	8.69e-53	143 – 180
0.80	Tracks – Squares	2.27e+03	9.03e-37	142 – 180
1.00	Control – Tracks	2.15e+04	3.61e-49	146 – 148
1.00	Control – Squares	2.86e+04	4.21e-55	146 – 197
1.00	Tracks – Squares	1.94e+03	3.44e-43	148 – 197
1.20	Control – Tracks	1.99e+04	5.30e-47	148 – 135
1.20	Control – Squares	2.86e+04	6.80e-56	148 – 194
1.20	Tracks – Squares	2.09e+03	1.77e-38	135 – 194
1.40	Control – Tracks	2.31e+04	3.28e-51	151 – 153
1.40	Control – Squares	3.09e+04	3.30e-58	151 – 205
1.40	Tracks – Squares	3.75e+03	7.49e-35	153 – 205
1.60	Control – Tracks	2.28e+04	6.73e-50	150 – 153
1.60	Control – Squares	2.86e+04	1.32e-56	150 – 191
1.60	Tracks – Squares	6.78e+03	1.35e-17	153 – 191
1.80	Control – Tracks	2.45e+04	7.60e-53	155 – 158
1.80	Control – Squares	3.17e+04	9.03e-59	155 – 205
1.80	Tracks – Squares	7.61e+03	4.84e-18	158 – 205
**Experimental**				
**DIV**	**comparison**	U **statistic**	p**-value**	N–N
7	Control – Tracks	5.59e+03	4.67e-21	37 – 151
7	Control – Squares	1.39e+04	1.27e-20	37 – 389
7	Tracks – Squares	2.66e+04	8.39e-02	151 – 389
10	Control – Tracks	7.26e+03	5.82e-20	32 – 227
10	Control – Squares	1.05e+04	1.51e-09	32 – 398
10	Tracks – Squares	2.29e+04	1.23e-24	227 – 398

Table 2. Linear regression model output for Fig. 2D. Ordinary least squares model output predicting burst sizes from average axon length $\langle \ell \rangle$, condition, and their interaction.

**Model**	
Model:	coactivation size $\sim \langle \ell \rangle$ + condition + $\langle \ell \rangle \times$ condition
Number of observations:	4291
Degrees of freedom (model):	5
Degrees of freedom (residuals):	4285
Statistics:	R^2: 0.768, adjusted R^2: 0.768
	F: 2840, Log-Likelihood: 3296.4, AIC: -6581, BIC: -6543

**Parameters**						
**parameter**	**slope**	**standard error**	**t-statistic**	**p-value**	[**0.025**	**0.975**]
intercept	0.9522	0.007	138.045	0.000	0.939	0.966
Tracks intercept	-0.6932	0.010	-71.883	0.000	-0.712	-0.674
Squares intercept	-0.3016	0.009	-31.998	0.000	-0.320	-0.283
$\langle \ell \rangle$	0.0292	0.006	4.835	0.000	0.017	0.041
$\langle \ell \rangle$: *Tracks*	0.2180	0.008	25.765	0.000	0.201	0.235
$\langle \ell \rangle$: *Squares*	0.1007	0.008	12.329	0.000	0.085	0.117

Table 3. Statistics for Fig. 3C. Statistics for the two-sample Mann-Whitney-Wilcoxon tests indicated in Fig. 3C.

**comparison**	U **statistic**	p**-value**	N–N
Control – Tracks	1.10e+04	6.59e-36	109 – 101
Control – Squares	2.16e+02	4.13e-33	109 – 108
Tracks – Squares	1.18e+04	4.82e-37	101 – 108

Table 4. Statistics for Fig. 3D. Statistics for the two-sample Mann-Whitney-Wilcoxon tests indicated in Fig. 3D across different noise levels σ. Non-significant ($p \geq 0.05$) comparisons are highlighted in red.

h	**comparison**	U **statistic**	p**-value**	N–N
0.0	$\sigma = 1.75 - \sigma = 2.00$	5.41e+03	9.87e-12	64 – 104
0.0	$\sigma = 2.00 - \sigma = 2.25$	8.51e+03	1.56e-03	104 – 132
0.0	$\sigma = 1.75 - \sigma = 2.25$	7.37e+03	2.80e-17	64 – 132
0.1	$\sigma = 1.75 - \sigma = 2.00$	3.18e+03	4.88e-01	59 – 101
0.1	$\sigma = 2.00 - \sigma = 2.25$	5.60e+03	7.28e-01	101 – 114
0.1	$\sigma = 1.75 - \sigma = 2.25$	3.61e+03	4.22e-01	59 – 114
0.3	$\sigma = 1.75 - \sigma = 2.00$	3.70e+03	3.59e-01	67 – 102
0.3	$\sigma = 2.00 - \sigma = 2.25$	4.45e+03	6.15e-04	102 – 119
0.3	$\sigma = 1.75 - \sigma = 2.25$	3.14e+03	1.60e-02	67 – 119
0.5	$\sigma = 1.75 - \sigma = 2.00$	2.63e+03	1.49e-03	72 – 102
0.5	$\sigma = 2.00 - \sigma = 2.25$	5.46e+03	1.66e-01	102 – 120
0.5	$\sigma = 1.75 - \sigma = 2.25$	2.60e+03	4.16e-06	72 – 120

Table 5. Statistics for Fig. 3D. Statistics for the two-sample Mann-Whitney-Wilcoxon tests indicated in Fig. 3D across different heights h. Non-significant ($p \geq 0.05$) comparisons are highlighted in red.

comparison	U statistic	p-value	N–N
$\{\sigma = 1.75, h = 0.1\} - \{\sigma = 1.75, h = 0.3\}$	2.01e+03	8.87e-01	59 – 67
$\{\sigma = 2.00, h = 0.1\} - \{\sigma = 2.00, h = 0.3\}$	5.20e+03	9.02e-01	101 – 102
$\{\sigma = 2.25, h = 0.1\} - \{\sigma = 2.25, h = 0.3\}$	5.30e+03	3.97e-03	114 – 119
$\{\sigma = 1.75, h = 0.3\} - \{\sigma = 1.75, h = 0.5\}$	2.56e+03	5.23e-01	67 – 72
$\{\sigma = 2.00, h = 0.3\} - \{\sigma = 2.00, h = 0.5\}$	3.50e+03	5.36e-05	102 – 102
$\{\sigma = 2.25, h = 0.3\} - \{\sigma = 2.25, h = 0.5\}$	6.14e+03	6.12e-02	119 – 120
$\{\sigma = 1.75, h = 0.1\} - \{\sigma = 1.75, h = 0.5\}$	2.33e+03	3.37e-01	59 – 72
$\{\sigma = 2.00, h = 0.1\} - \{\sigma = 2.00, h = 0.5\}$	3.49e+03	7.07e-05	101 – 102
$\{\sigma = 2.25, h = 0.1\} - \{\sigma = 2.25, h = 0.5\}$	4.32e+03	1.12e-06	114 – 120

Table 6. Statistics for Fig. 3E. Statistics for the two-sample Mann-Whitney-Wilcoxon tests indicated in Fig. 3E across different noise levels σ.

h	comparison	U statistic	p-value
0.0	$\sigma = 1.75 - \sigma = 2.00$	0.00e+00	1.61e-04
0.0	$\sigma = 2.00 - \sigma = 2.25$	0.00e+00	1.58e-04
0.0	$\sigma = 1.75 - \sigma = 2.25$	0.00e+00	1.57e-04
0.1	$\sigma = 1.75 - \sigma = 2.00$	0.00e+00	1.62e-04
0.1	$\sigma = 2.00 - \sigma = 2.25$	0.00e+00	1.50e-04
0.1	$\sigma = 1.75 - \sigma = 2.25$	0.00e+00	1.53e-04
0.3	$\sigma = 1.75 - \sigma = 2.00$	0.00e+00	1.39e-04
0.3	$\sigma = 2.00 - \sigma = 2.25$	0.00e+00	1.49e-04
0.3	$\sigma = 1.75 - \sigma = 2.25$	0.00e+00	1.51e-04
0.5	$\sigma = 1.75 - \sigma = 2.00$	0.00e+00	1.43e-04
0.5	$\sigma = 2.00 - \sigma = 2.25$	0.00e+00	1.49e-04
0.5	$\sigma = 1.75 - \sigma = 2.25$	0.00e+00	1.50e-04

Table 7. Linear regression model output for Fig. 3E. Ordinary least squares model output predicting number of bursts from obstacle height h and noise level σ. Non-significant ($p \geq 0.05$) comparisons are highlighted in red.

**Model**	
Model:	# of bursts $\sim h + \sigma$
Number of observations:	120
Degrees of freedom (model):	2
Degrees of freedom (residuals):	117
Statistics:	R^2: 0.907, adjusted R^2: 0.905
	F: 568.3, Log-Likelihood: -277.84, AIC: 561.7, BIC: 570.1

Parameters

parameter	slope	standard error	t-statistic	p-value	[0.025	0.975]
intercept	-42.4520	2.247	-18.890	0.000	-46.903	-38.001
h	-1.3616	1.180	-1.154	0.251	-3.698	0.975
σ	37.4000	1.110	33.693	0.000	35.202	39.598

Table 8. Statistics for Fig. 4B. Statistics for Kologorov-Smirnov tests between distributions.

**comparison**	D **statistic**	p**-value**	N–N
Control – Tracks	1.58e-01	0.00e+00	$1.57e+06-9.35e+05$
Control – Squares	1.32e-01	0.00e+00	$9.35e+05-1.16e+06$
Tracks – Squares	4.03e-02	0.00e+00	$1.57e+06-1.16e+06$

Table 9. Statistics for Fig. 4D. Statistics for Kologorov-Smirnov tests between distributions.

**comparison**	D **statistic**	p**-value**	N–N
Control – Tracks	5.86e-01	0.00e+00	$2.83e+04-2.83e+04$
Control – Squares	3.26e-01	0.00e+00	$2.83e+04-2.83e+04$
Tracks – Squares	2.60e-01	0.00e+00	$2.83e+04-2.83e+04$

Table 10. Statistics for Fig. 4E. Results of two-sample Student's t-test between different conditions and for each condition versus null distribution, per measure.

**Between conditions**			
**measure**	**comparison**	D **statistic**	p**-value**
global efficiency	Control – Tracks	3.17e+02	3.51e-35
global efficiency	Control – Squares	1.46e+02	4.07e-29
global efficiency	Tracks – Squares	-1.07e+02	1.15e-26
modularity Q	Control – Tracks	-6.24e+01	1.74e-22
modularity Q	Control – Squares	-3.67e+01	2.24e-18
modularity Q	Tracks – Squares	3.21e+01	2.44e-17
avg. clustering	Control – Tracks	3.92e+02	7.56e-37
avg. clustering	Control – Squares	1.34e+02	1.79e-28
avg. clustering	Tracks – Squares	-6.95e+01	2.46e-23
**Condition versus null**			
**measure**	**comparison**	D **statistic**	p**-value**
global efficiency	Control – null	9.84e+03	4.94e-62
global efficiency	Tracks – null	2.58e+03	1.39e-51
global efficiency	Squares – null	2.44e+03	4.04e-51
modularity Q	Control – null	2.65e+02	8.65e-34
modularity Q	Tracks – null	3.31e+02	1.66e-35
modularity Q	Squares – null	3.73e+02	1.86e-36
avg. clustering	Control – null	2.15e+03	3.92e-50
avg. clustering	Tracks – null	6.55e+02	7.51e-41
avg. clustering	Squares – null	3.98e+02	5.74e-37
